# How to Assess Ictal Consciousness?

**DOI:** 10.3233/BEN-2011-0316

**Published:** 2011-03-29

**Authors:** Mirja Johanson, Katja Valli, Antti Revonsuo

**Affiliations:** ^1^Neurological Rehabilitation ClinicStora Sköndal FoundationSköndalSweden; ^2^Centre for Cognitive NeuroscienceDepartment of Behavioural Sciences and PhilosophyUniversity of TurkuTurkuFinland; ^3^School of Humanities and InformaticsUniversity of SkövdeSkövdeSweden

**Keywords:** Epilepsy, seizures, ictal, level of consciousness, content of consciousness, Phenomenology of Consciousness Inventory (PCI), Ictal Consciousness Inventory (ICI), Responsiveness in Epilepsy Scale (RES), EpiC (Epilepsy-specific Content analysis of contents of consciousness in partial seizures)

## Abstract

Despite the complexity and methodological difficulties in defining the concept of consciousness, it is a central concept in epileptology, and should thus be tractable for scientific analysis. In the present article, a two-dimensional model consisting of concepts related to the level and the contents of consciousness will be presented. This model has been found to be well suited for the description of seizure-induced alterations of consciousness, and is supported both by findings from neuroimaging and electrophysiological studies as well as from phenomenological studies. Further, we will review both traditional introspective methods as well as methods that have recently been developed or utilized in epilepsy research, summarize the main findings concerning first person experiences during epileptic seizures acquired with some of these methods, and discuss their strengths and weaknesses.

## 1. Introduction

Although consciousness is a central concept in many research fields, there is no generally agreed definition of it. It has been emphasized that the essence of consciousness and what makes the mind-body problem intractable lies in the subjective experience [45] also called "phenomenality", or "phenomenal consciousness" [8].

In epileptology, the term consciousness plays a central role because according to the international classification of epileptic seizures, simple and complex partial seizures are distinguished by impaired consciousness [14]. Impaired consciousness is defined as "the inability to respond normally to exogenous stimuli due to altered awareness and/or responsiveness" [22]. Nonetheless, as the concepts 'consciousness' and 'awareness' have not been defined in more detail, the concepts of impaired awareness, and thus 'consciousness', remain unclear [22].

Another problem is that responsiveness may be dissociated from phenomenal consciousness. Such a dissociation has been reported during general anaesthesia and called "anaesthesia awareness" or "anaesthesia dreaming" (the person looks nonconscious but can have dream-like experiences or memories of what happened during anaesthesia) [17,41]. Automatic behavior in complex partial seizures includes the converse dissociation between consciousness and behaviour (the person looks conscious but has no, or at least later does not recall any, subjective experiences). Consequently, responsiveness does not necessarily indicate the presence of subjective experience, and lack of responsiveness does not necessarily indicate the absence of subjective experience [54].

## 2. Conceptual tools for the study of consciousness in epileptic seizures

Epileptic seizures affect both the level of consciousness and the patient's subjective experiences, that is,

ISSN 0953-4180/11/$27.50 © 2011 – IOS Press and the authors. All rights reserved

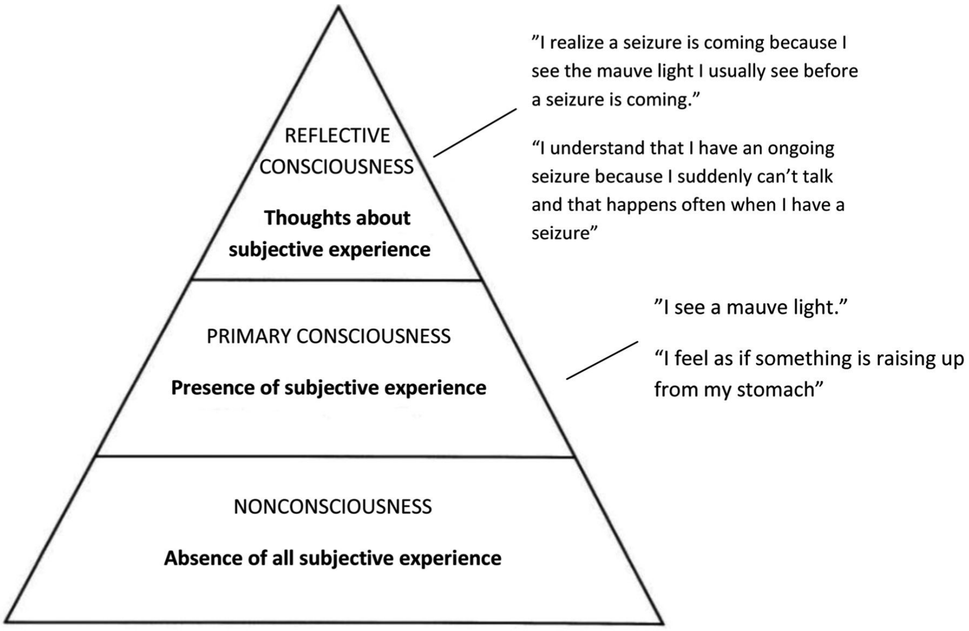

Fig. 1. Farthing's model of levels of consciousness and examples of seizure related experiences as categorized into belonging to different levels of consciousness.

contents of consciousness. The level of consciousness is affected especially in generalized tonic-clonic seizures and in absence seizures whereas the contents of phenomenal consciousness are affected in focal seizures. Sometimes both are affected, as can be the case in temporal lobe seizures.

A two-dimensional model including the distinction to the *level* and the *contents* of consciousness can be valuable in assessing the qualitative and quantitative changes in consciousness in connection with seizures. The distinction between the level and contents of consciousness was first presented by Plum and Posner [51] and the importance of a bi-phasic model for the understanding of different kind of seizures has been later emphasized by Gloor [20]. Not until recently, however, concrete models based on the distinction have been introduced and found to be useful both for theoretical and clinical purposes in the study of epilepsy [13,30].

For categorization of (*1*) *the levels of consciousness*, Farthing's [18] model has to do with the different degrees of availability or retrievability of contents (See Fig. 1). The levels of consciousness in Farthing's model can be related to different phases of partial epileptic seizures as follows: (1) A reported total absence of subjective experience is categorized as nonconsciousness, (2) If the person remembers having had any kind of subjective experiences at any time during the seizure, the patient is assumed to have had primary consciousness present during seizure, (3) If the patient became aware of a forthcoming seizure before the beginning of it, or became aware of an ongoing seizure (thus having had thoughts about the subjective experience related to seizure), the patient is assumed to have had reflective consciousness during seizure (Fig. 1).

(*2*) *The contents of consciousness* can be categorized with various methods, and are usually grouped under major categories comprising majority of our subjective experiences. For example, in Pekala's [47] Phenomenology of consciousness Inventory (PCI), a method devised for the study of potentially any state of consciousness, he divided subjective experiences into the following main categories: sensations, perceptions, emotions, thoughts and images. This and other methods for assessing the content of experiences during seizures will be presented later.

The analysis of the levels and contents of consciousness can be completed by the description of (*3*) *the temporal progression of seizure* (*see Fig. 2*), by joining these two. This enables us to tie different aspects of the seizure process together. The descriptions of the progress of the seizure (from the patient and/or from a significant other) can usually be divided into three phases:

1) the descending period characterized by a descending level of consciousness

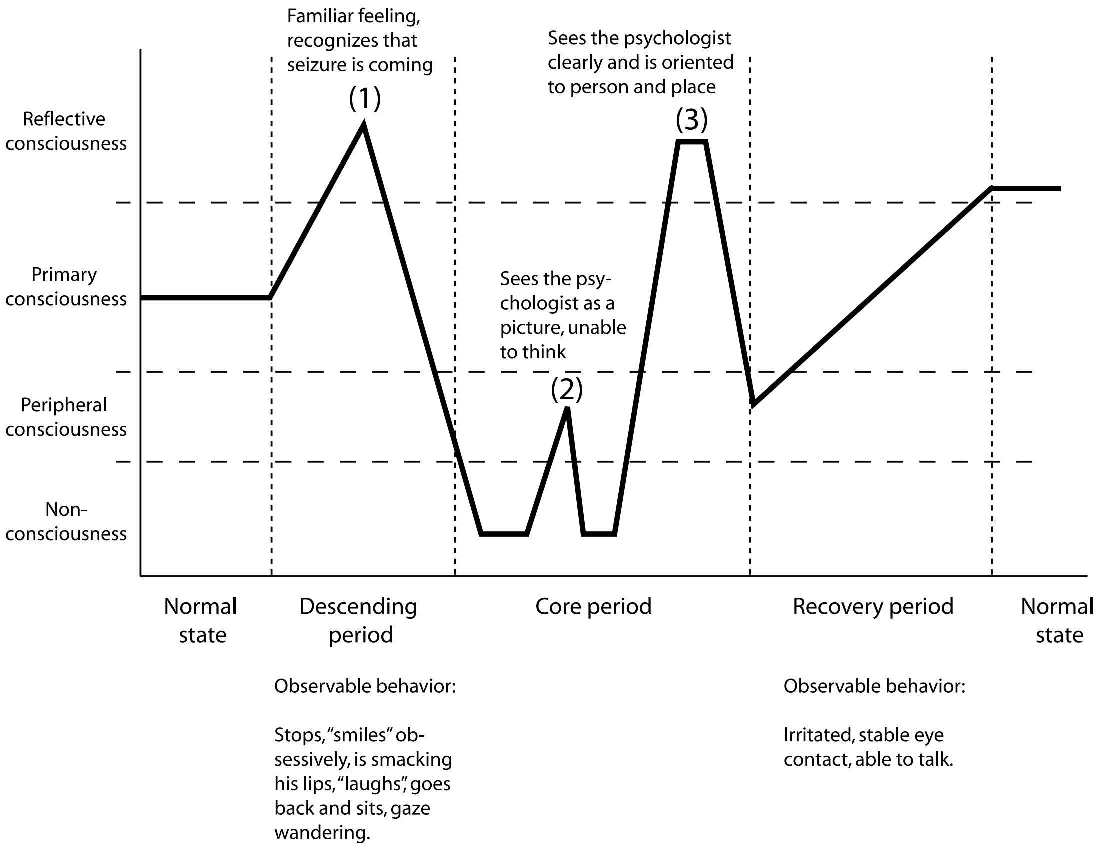

Fig. 2. A schematic illustration of the levels, contents and behavior of one patient during a neuropsychological assessment (Modified from Johanson et al. [30]).

2) the core period characterized by the most severe disturbances of consciousness
3) the recovery period characterized by an ascending level of consciousness and return to the normal state.

An overall picture of the seizure is valuable and helps both the researcher and the patient to understand the seizure phenomenon in more detail.

Monaco et al. [42,43] have described another two-dimensional model, also distinguishing between the level and the contents of consciousness. The model aims to describe alterations of consciousness during the normal state, during generalized seizures, and during simple and complex partial seizures. The first dimension, the level of consciousness is a range of conscious and unconscious states extending from alert wakefulness through sleep into coma. Video monitoring has been used to document the full extent of ictal unresponsiveness as a testable measure of the level of consciousness. The second dimension of consciousness is the content of subjective experience: sensations, emotions, memories, intentions and feelings.

Figure 3 illustrates the level and contents of consciousness in a biaxial diagram, in which dots indicate the possible conscious states. In a healthy subject, the level of arousal is almost constantly high and the vividness of the contents in the wakeful state shows wide variation. During generalized seizure both the level and contents of subjective experience are absent. The altered conscious state in focal seizure with experiential contents consists of a great variation in the level of awareness and almost constant vivid contents of consciousness whereas in limbic status epilepticus the level of consciousness may vary but subjective experiences are absent.

Separate neural mechanisms have been shown to underlie the level and the contents of consciousness and therefore neurophysiological and other functional brain imaging findings provide a good basis for this model [12]. Impairment of the general level of awareness seems to relate to primary (generalized seizures) or

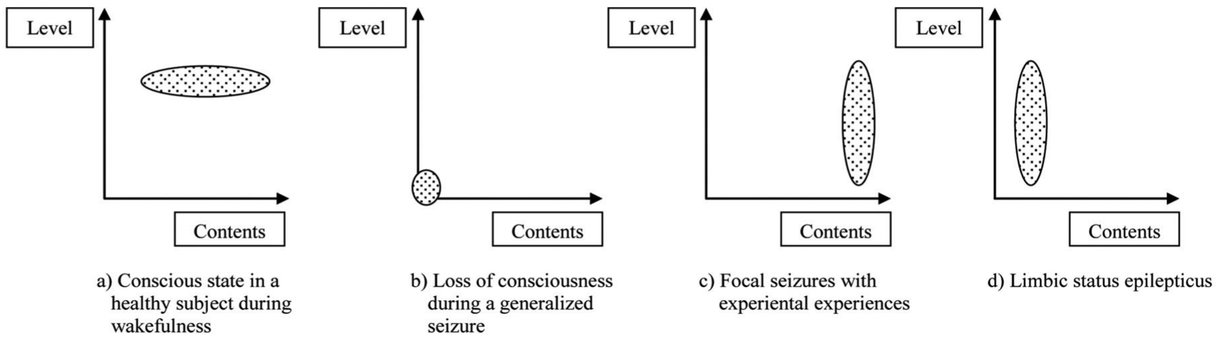

Fig. 3. Illustrations of the bidimensional model of consciousness (Modified from Cavanna and Monaco [12,13]).

secondary (partial seizures) involvement of subcortical structures [4]. This leads to transient disruption of frontoparietal and midline interhemispheric associative networks, according to the 'default mode' of brain function (a suggested existence of an organized baseline state of neural activity). The qualitative aspects of experiential phenomena (contents of consciousness), on the other hand, are mainly a consequence of the activity of limbic components of the temporal lobe [12]. The two dimensions as a discriminator between the level and contents of consciousness became also obvious when tested on patients with temporal lobe epilepsy. The results demonstrated that even if the level and content scores were positively correlated, only the content scores correlated significantly with mood and anxiety disorders [44]. Interestingly, preliminary results show differences in subjective reports of conscious experiences between patients who have epilepsy and patients with non-epileptic seizures but it is too early to confirm the evidence of subjective seizure experiences for differential diagnosis [3].

## 3. Practical tools for assessing ictal consciousness

### 3.1. Introspective and retrospective methods

Descriptive introspection is a process where we choose to focus our attention on particular contents of phenomenal consciousness and then formulate a verbal report concerning the experience. During introspection we use reflective consciousness with the intention to deliberately observe the contents of primary consciousness [18,54].

Introspection has serious limitations but is still the best method for studying consciousness and the mind [18,54]. There are general methodological limitations related to the use of introspection and verbal reporting, and in epilepsy research, additional limitations complicate data collection. Generally subjective experiences reported introspectively can be influenced, for example, by forgetting, filling in gaps from other seizures, and reconstruction of memories [38,62]. It is therefore important to collect the reports as soon as possible after seizure and to instruct the subject that he/she is supposed to report absolutely *everything* that he/she experienced, exactly as he experienced it, without leaving anything out and without adding anything. Some experiences may be *ineffable* [28]; even if clearly recalled, experiences may be so bizarre or so unusual that there are no words in ordinary language to describe them [31]. Some subjects may be inclined to *censor* some of their experiences, especially those of sexual or violent nature. This problem may be overcome by guaranteeing total anonymity in the research and/or creating a safe, accepting and supportive atmosphere. Furthermore, even the intention to observe and the process of observation of one's experiences might change the experiences from what they would have been naturally. Thus, the task of reporting one's seizure experiences may actually influence the content of seizures [55]. Finally, whatever the subjects report, there is no way in which the accuracy of the reports could be independently checked or verified. We have to rely only on the subjective report. Several reviews agree that introspective data can be both valid and useful [15,18,36,39]. Singer and Kolligan [61] state that "self-report questionnaires all converge in suggesting that people can generally provide reasonably valid and reliable indices of their own differential patterns of ongoing thought).

#### 3.1.1. Thinking-out-loud

The *Thinking-out-loud*-method (or *direct introspection*) uses direct online introspective reporting. Using this method, the patient should verbally express, aloud and in real time, everything that happens in consciousness. Applied to epilepsy, this method could be used to express and record especially the verbal inner speech in reflective consciousness during a seizure. Thinking-out-loud method, at least in modified form, has been used during electrical brain stimulation, often combined with interview techniques [50,65].

This method enables the description of the actual/ongoing stream of consciousness and is therefore not affected by memory problems in the same way as methods which are based on reporting from memory. It can be used when the contents of consciousness are distorted but the level of consciousness is intact, at least in some degree, as is the case during simple partial seizures and in the beginning of complex partial seizures. The problem with this method is that *having* an experience and *reporting* the experience simultaneously easily interfere with each other, because it is very difficult to do both at the same time. This seems to be the case for many patients with epilepsy, as during seizure most patients have difficulties in controlling the direction of attention (which turns inwards, and the focus of attention narrows during seizure start) [6,30,31]. Thus, this method could be used only with a minority of patients, preferably those who have a good knowledge of their experiences, who's experiences are similar from seizure to seizure, who are motivated, skilled and experienced in giving verbal introspective reports of their experience, and who retain attention control during seizure.

### *3.1.2. Questionnaire*

Questionnaires have often standardized answers that make it simple to compile large amounts of data from large amounts of participants in relatively short period of time. One limitation is that questionnaires are often closed in the sense that people can only give answers that fit into a question-and-response format. Thus, if using *only* inventories, researchers cannot discover anything that is totally different from the possibilities that they had anticipated. Second, considering the possibility of how cognitive problems may affect the filling in of a questionnaire, inventories are also sharply limited by the fact that respondents must be able to understand the relativity of a scale (e.g. visual scale), and to be able to read the questions and respond to them. On the other hand, questions and statements may help the subject to find words for his/her experiences which may be otherwise difficult when expressing seizure experiences.

Ronald J. Pekala [47] is one of the few researchers to have developed systematic questionnaire methods for quantifying consciousness and its various states. The methodology he devised, especially the retrospective self-report inventory 'Phenomenology of Consciousness Inventory' (PCI), helps to quantify and statistically assesses the variables associated with phenomenological experience. For research purposes it still is probably the most flexible and best documented instrument to evaluate different states of consciousness [48]. The PCI consists of 53 questions dealing with attention, affect, imagery, volitional control, altered states of consciousness, altered experience, self-awareness, memory and arousal. Extensive calculations strongly indicate acceptable reliabilities for the (sub)dimensions of the PCI [47]. It has been found to reliably and validly map states of consciousness in several contexts for instance, hypnosis [37,47], out-of-body experience [5], near-death event, NDE [21] and meditation [64]. It has also been tested for people with schizophrenia [57] and recently for people with partial epileptic seizures [31].

The PCI was found well suited for the assessment of the contents of consciousness in connection with partial seizures, especially for comparisons between epilepsy and other altered states of consciousness, as well as for comparisons between the normal state of consciousness and consciousness during seizures in the same individuals. Some of the PCI items, however, can be regarded as abstract, academic and complicated. This is why modifications of the PCI are recommended for clinical use and that the participants always complete the inventory together with the researcher. However, the PCI is a valuable instrument with motivated patients who have an interest, and ability, in self-reflection.

Recently, another self-report measure on patients' experiences concerning altered conscious states during epileptic seizures has been presented. This Ictal Consciousness Inventory [ICI;13] is specifically created for investigation of ictal conscious state of patients with temporal lobe epilepsy. The ICI is short in form and the questions are easy to understand. It is a 20-item questionnaire generated on the basis of interviews with patients, literature review, and consultation with experts. It is aimed to the quantification of the level of general awareness/responsiveness (the first ten items) and the "vividness" of ictal experiential phenomena (the last ten items). This inventory fullfills the standard criteria for acceptability, reliability, and face and construct validity. It also validates a clinically valuable distinction between the level and content of consciousness by indicating that they are related to different neural functions [13].

Furthermore, even more recently, a new tool for prospectively evaluating impaired consciousness during seizures has been created [67]. This standardized evaluation battery, the Responsiveness in Epilepsy Scale (RES), measures responsiveness during epileptic seizures and is designed for use in patients during continuous video/EEG monitoring. It assesses some sensory, motor, and memory functions during and following seizures. Initial reliability testing (test-retest and interrater reliability) of normal subjects with simulated seizures are in acceptable range [67].

### 3.1.3. *Interview*

There are different types of interview methods. A *structured interview* has a formalized, limited set questions and the aim is to ensure that each interview is presented with exactly the same questions in the same order. The choice of answers to the questions is often fixed in advance, though open-ended questions can also be included. A *semi-structured interview* is flexible, allowing new questions to be brought up, and to adjust the questioning style to suit for different types of participants. The freedom to tailor questions is an important advantage in epilepsy research where patients'experiences differ between individuals. Some patients also need help in organizing their experiences because of cognitive deficits [1,2,66] and need support because of frequent negative emotions that are associated to seizure-related experiences [24,31].

*Unstructured interview* advocates listening to how each subject responds to the question, and then the interviewer can change or adapt the subsequent questions to meet the respondent's understanding or beliefs. It enables the researchers to add questions beyond what they had planned in advance, and it enables them to clarify the meaning of the responses they receive. The advantages are that the collected data is an exact account of what the interviewee has said, the information is about the real life episode (episodic knowledge) the subject lived through, and the subject is neither guided by questions or restricted by them. The researcher can also find new important information and can cope with sensitive issues as the subject is treated as a unique individual. This is a considerable advantage when collecting seizure reports which may include bizarre and peculiar experiences, for example, out-of-body experiences, misperceived or real memories, feelings of depersonalization and other hallucinatory experiences [31].

One problem with systematic analysis of spontaneous seizure reports has been the lack of suitable methods. However, a new method has recently been developed for this purpose. It is modified from a content analysis method used in dream research [56] and is called EpiC, the Epilepsy-specific Content analysis of contents of consciousness in partial seizures [31]. Content analysis is a method by which we can convert verbal, written or other symbolic material into numbers so that statistical analysis can be performed [53]. This method is reliable but quite time consuming. When using EpiC the analysis of seizure descriptions is carried out in two stages. First, the description of the actual subjective experience and seizure-related behaviour is identified. Second, the identified experiences and behaviour are classified with regard to specific content. Both stages are carried out by two raters independently and interrater agreement is calculated. At least 80% agreement is necessary for reliable results. A more detailed description of the content analysis method is presented in Johanson et al. [31].

### 3.1.4. *Diary*

Diaries are written narrative reports on a person's activities and thoughts over a period of time. The subjects are given the instructions to write down everything they experienced in connection with the seizure process, exactly the way they experienced it as soon as possible after the seizure to avoid memory bias. The diary method can be used to collect seizure experiences for a selected group of patients. This method is more demanding for the subject than oral reporting, as it requires more effort, a good executive ability, higher level of attention, self-confidence and adequate verbal ability to be able to express oneself in writing. After seizure, experiences of tiredness, negative feelings and cognitive impairment are common [11,19,33,34], which increases the effort required in intellectual tasks. Because diaries include unsystematic reports collected under uncontrolled conditions (no matter how well the participants are instructed in advance), they may not be useful for rigorous testing of hypotheses [18]. However, the usefulness of diaries for systematic research, such as studying the seizure process, can be increased by teaching subjects to keep systematic seizure diaries.

Seizure diaries have traditionally been used for collecting information about the frequency of seizure occurence. However, also diaries including the patient's own seizure history, seizure incidence associated with premonitory symptoms and risk factors for seizures, have been studied, for example, to predict subsequent seizures [25]. The diary method has turned out to be both a data collecting tool and also an educative instrument which also may reduce seizure frequency [23]. Paper and pencil diaries are most frequently used but electronic data collection is increasing.

Table 1
Examples of studies presenting subjective experiences preceding or in connection with epileptic seizures. PS = partial seizures, SPS = simple partial seizures, CPS = complex partial seizures GS = generalized seizures, PGS = primarily generalized seizures, DCPS = during conscious part of seizure (SPS and start of CPS)

| Author | Patients and methods | Focus of the study | Frequent experiences $\geqslant$ 10 % | Less frequent experiences $<$ 10 % |
|---|---|---|---|---|
| Hughes et al. 1993 | 148 adult patients<br>128 PS, 20 GS<br>Semi-structured interview | Premonitory symptoms | Irritability 25%<br>Headache 13%, Depression 10% | Funny feeling 8.3%, confusion and anxiety 6.7% each, fear, elation, speech disturbances, polyuria and menstrual symptoms 3.3% each, anger, childish behavior, urge to defecate, limb weakness and trembling 1.7% each |
| Schulze-Bonhage et al. 2006 | 500 adult out-patients<br>415 PS, 82 PGS<br>Questionnaire | Premonitory symptoms | | Nausea, impaired concentration, dizziness and tiredness 4% each, restlessness 2%, headache 1.2%, malaise 1% |
| Rajna et al. 1997 | 562 in-patients, all type of seizures, 8–69 years, Questionnaire | Frequency of warning and initial symptoms | Headache, epigastric sensation, funny feeling | Fatigue |
| Janszky et al. 2004 | 100 patients, medial temporal lobe lesions<br>Semi-structured interview | Isolated auras | Abdominal 69%, affective 25%, dysmnestic 15% | Somatosensory and vertigo 6% each, olfactory 5%, auditory 3%, gustatoric 1% |
| Tuxhorn 2005 | 75 adult (of 600) patients<br>Retrospective analysis of video EEG monitoring | Somatosensory auras, features of seizure semiology predictive for localization | Tingling 77 % | Sense of pulling 8%, cold/heat 5%, numbness 5%, pain 2%, sense of movement 1% |
| Silberman et al. 1994 | 21 adult patients<br>19 GP<br>15 CPS<br>Retrospective structured interview | Aura and psychopathology, lifetime occurrence of subjective and behavioral symptoms | Thought and speech disturbances 100%, motor automatisms 86%, hallucinations 71%, sensory illusions and distortions 33%, cognitive illusions 24% (e.g. déjà vu), paroxysmal affect 14% (e.g.fear, euphoria, sexual) | Time distortions |
| Erkwoh & Steinmeyer 1996 | 325 inpatients, 27% CPS, 24% GS, 4.5% other focal seizures, 29.5% mixed CPS and GS, Medical records | Phenomenology of SPS | Autonomous 16% (warmth, sensations from stomach, chest, often raising up), visual 10% (e.g. blurred vision, diplopia, optic hallucinations) | Dreamy state 5%, sensory 4% (e.g. prickling, tingling, "electrical", pain), olfactory/gustatory 3%, vestibular 0.8%, ictal fear 0.6%, auditory 0.3% |
| Johanson et al. 2008 | 40 patients, CPS, 262 seizure descriptions<br>Epilepsy-specific content analysis of contents of consciousness, EpiC | To describe the qualitative features of subjective experiences in PS with a new method, EpiC | Sensations 98% (DCPS: modality-specific 55% (most common visual,ineffable 16%), bodily 63% (most common visceral), hallucinatory36%, (most common distortion of reality)), (passive) events 52%, voluntary actions 56%, thinking 60%, emotions 50% (fear and discomfort most frequent DCPS; sadness, depression and fear most common afterwards), cognition 36% of seizure reports, reflective consciousness 45% | DCPS: modality-specific sensations: tactile, olfactory, taste;<br>bodily sensations: feeling of cold/ heat, restlessness<br>emotions: negative feeling of losing control, sadness, depression, anger, frustration, joy, positive feeling of having control<br>After seizure: few modality-specific sensations 6%, general confusion 7% |

Questionnaires [59], retrospective structured [60] and semi-structured interviews [27,29] as well as analysis of medical records [16], of video EEG monitoring [63] and stereoelectroencephalographic recording [65] have been used in collecting data about seizure-related subjective contents of consciousness (See Table 1 for more details).

### 3.2. Observation

Some patients with epilepsy can engage in quite complex actions during seizures without being aware of their own behavior. Several descriptions of zombie-like behavior have been presented in the literature for example Dr Z, [35,49]. However, it is difficult to verify objectively whether the patients are or are not having subjective experiences during the automatic behaviours. We can only verify that they have no memories of experiences from that period, but subjectively, total amnesia and total nonconsciousness seem retrospectively the same, as a total blank in memory. Thus, in epilepsy research it is important to try to discriminate between the loss of consciousness and the loss of memory. First, according to the terminology defined earlier, true loss of consciousness involves having no phenomenal consciousness at all, not even in an altered form. Second, the only way for us to know whether the patient has had phenomenal consciousness present during the behavior is the subjective retrospective report delivered by the patient. Consequently, we have no means of checking whether or not the patient is truly nonconscious during the automatic behavior.

Furthermore, lack of any recollection of the seizure is a necessary but not sufficient criterion to show that phenomenal consciousness was absent. If the patient remembers that she/he lost consciousness before losing memory and describes regaining consciousness and also describes the period of memory gap as a total blackout, then we can assume that she/he was to all intents and purposes nonconscious and that the state of nonconsciousness caused the amnesia. But, of course, there is still no absolute guarantee that this was the case. In addition, there are patients who have "pure amnestic seizures". During this kind of seizures the patient's behavior is totally normal, including for example a conversation with somebody. There seems to be no loss of consciousness, but the patient has a total amnesia for the episode [46]. Consequently, during pure amnestic seizures the memory encoding of experiences is absent but we have no reason to believe that the experiences themselves would be absent [54].

Also patients suffering from severe global amnesia report that they often feel as if they just awoke from a lengthy period of total unconsciousness (e.g. the famous case of Clive Wearing) [35].

Because of loss of consciousness and/or memory, information from somebody who has witnessed the seizure-related behavior is needed. However, it seems that reporting seizure behaviors in detail is not an easy task. For evaluation of the accuracy of patient and witness's observations, their accounts have been compared with observations made by professionals after watching videotaped seizures. Structured questionnaires [58] (including assessment of level of consciousness) as well as open format have been used [40]. Mannan [40] shows that questionnaire terminology can be difficult to interpret and also fails to take into account the evolution of seizure, and that is why a more open format isto be preferred. Studies confirm that inaccuracies are common in observations; language disturbances, complex automatisms, and autonomic signs are obvious and easy to memorize whereas dystonic posturing and upper limb distal automatisms are more frequently missed [58]. Facial expressions are frequently reported by witnesses [7] in contrast with hand automatisms and motionless staring [7,26].

## 4. Discussion

An empirically based scientific field must start with careful description of the phenomenon to be explained. Knowing what it is like to have subjective experiences related to a certain clinical condition is the key for understanding that condition. Consequently, measuring ”What it is like to have a seizure” is one of the core questions to be answered for increased understanding of epilepsy.

There is a general agreement that both the internal (subjective experience) and external (behavior) standpoint must be considered when studying consciousness. Therefore both patient and witness reports are necessary as a starting point for describing ictal consciousness. However, there has been a lack of systematic studies concerning the quality of phenomenal experiences during epileptic seizures. One reason for this is probably that the field has suffered both from conceptual and methodological problems. During recent years, an increasing amount of papers have been published concerning the clarification of concepts, creating new methods and further understanding of the neural correlates of ictal consciousness [10,12,68].

Concerning the conceptual framework, during recent years several research groups have concluded that a two-dimensional model based on the distinction between the level and the contents of consciousness is to be preferred in the comprehensive assessment of ictal consciousness [9,13,44]. The model is supported by neuroimaging, electrophysiological and phenomenological evidence.

Recently, inventories specifically developed for investigation of ictal consciousness for both research and clinical use have been presented. The PCI questionnaire, which has been developed for the study of the structures and patterns in consciousness and allows them to be empirically quantified and statistically compared [47] has been tested in the field of epilepsy. In terms of validity, it is interesting to note that when the same seizures were assessed with two different methods (EpiC and PCI), they yielded similar results [32] although EpiC is based on content analysis of spontaneous seizure descriptions whereas PCI is a self-report inventory with predetermined items. The focus of ICI is temporal lobe seizures, and it covers both the level and the contents of consciousness. The new Responsiveness in epilepsy inventory, RES, is to be used to measure impaired consciousness during continuous video/EEG monitoring.

In sum, considerable progress has been made in studying the phenomenal aspects of epileptic seizures during the past ten years. The development of these methods and additional data collection will tremendously add to our knowledge of what is it like to have an epileptic seizure. This knowledge also has clinical use, as it will help the patients to understand their seizures and thereby also find better ways to cope with seizures and epilepsy.
